# Genetic diagnosis of a rare myrmecochorous species, *Plagiorhegma dubium* (Berberidaceae): Historical genetic bottlenecks and strong spatial structures among populations

**DOI:** 10.1002/ece3.4362

**Published:** 2018-08-07

**Authors:** Soo‐Rang Lee, Bo‐Yun Kim, Young‐Dong Kim

**Affiliations:** ^1^ Multidisciplinary Genome Institute Hallym University Chuncheon‐si Korea; ^2^ Department of Life Sciences Hallym University Chuncheon‐si Korea

**Keywords:** conservation, endangered species, genetic variation, microsatellite, migration rate, *Plagiorhegma dubium*, spatial structure

## Abstract

Distribution of genetic variation over time and space is relevant to demographic histories and tightly linked to ecological disturbances as well as evolutionary potential of an organism. Therefore, understanding the pattern of genetic diversity is a primary step in conservation and management projects for rare and threatened plant species. We used eight microsatellite markers to examine the level of genetic diversity, spatial structure, and demographic history of *Plagiorhegma dubium*, a rare myrmecochorous herb, populations sampled across northeast Asia and Siberia. We found low within‐population genetic variation associated with historical bottlenecks. Although pairwise *F*_ST_ values were not much higher than the ones found in similar life form species, STRUCTURE and PCoA revealed a clear broadscale spatial pattern of genetic structure. Bayesian clustering (best *K* = 6) and PCoA identified three populations that are distinctive from neighboring populations in the Korean peninsula, which suggests potential units for conservation and management plans in Korea. MIGRATE‐N and BAYESASS showed that both contemporary (0.003–0.045) and historical migration rates (2 × e^−5^−4.6 × e^−4^) were low. Our findings provide a good example, where genetic considerations should be integrated for conservation and management plans of rare and threatened species.

## 1 INTRODUCTION

Understanding genetic diversity patterns enables us to infer demographic changes driven by ecological disturbances in the past (Ellegren & Galtier, 2016; Ellstrand & Elam, 1993; Young, Boyle, & Brown, 1996). Since the emergence of human civilization, vast natural landscapes have been transformed to anthropogenically manipulated landscapes with regional habitat destructions (Lienert, 2004; Vitousek, Mooney, Lubchenco, & Melillo, 1997; Young et al., 1996). Although natural habitats are commonly patchy to some extent, habitat loss and fragmentation instantly shape populations to be very small and isolated. Thereby, habitat destruction accompanies a series of potential demographic processes, which in turn strongly influences not only genetic diversity but also spatial distribution of alleles and genotypes (Ellegren & Galtier, 2016; Ellstrand & Elam, 1993; Young et al., 1996). Despite growing interests, the influence of anthropogenic disturbances on genetic diversity remains relatively unexplored.

The most evident genetic consequences of habitat disturbances in plants are erosion of genetic variation and increased among‐population divergence resulted from drift, inbreeding, and limited gene flow (Ellegren & Galtier, 2016; Ellstrand & Elam, 1993; Young et al., 1996). Rare and endangered plant species with narrow distributions are subjected to genetic erosion driven by disturbed habitats (Awad, Fady, Khater, Roig, & Cheddadi, 2014; Jacquemyn, Roldán‐Ruiz, & Honnay, 2010; Ottewell, Bickerton, Byrne, & Lowe, 2015). For example, a microsatellite analysis revealed high inbreeding and recent population bottlenecks associated with habitat destruction in an extremely rare annual herb, *Rhinanthus osiliensis* (Aavik, Talve, Thetloff, Uuemaa, & Oja, 2017). The magnitude of genetic response to anthropogenic disturbances might depend on life history and ecological traits (Aguilar, Quesada, Ashworth, Herrerias‐Diego, & Lobo, 2008; Hylander & Ehrlén, 2013; Young et al., 1996). Negative effects of the disturbances on genetic diversity may be more pronounced in short‐lived plant species than in long‐lived trees (Young et al., 1996). Likewise, self‐incompatible outcrossing species are more prone to negative genetic effects of the disturbances than selfing plants because obligatory outcrossing plants often use clonal growth under severe disturbances (Honnay & Bossuyt, 2005; Honnay, Jacquemyn, Bossuyt, & Hermy, 2005).

Demographic processes following ecological disturbances may be inferred through examining patterns of genetic diversity using advanced tools of molecular analysis. For example, a molecular marker analysis revealed significantly lower heterozygosity and higher inbreeding in Pacific jumping mice (*Zapus trinotatus*), a riparian rodent, after a severe flood (Vignieri, 2010). Inference made through molecular markers is particularly useful for rare species with unknown demographic history as there is no need for destructive sampling. Furthermore, detailed information of demography and spatial distribution of genetic variation can also be applied to management plans for rare and endangered plant species. Using microsatellite data Straub and Doyle (2009) suggested two genetically distinct management units for the endangered legume (*Amorpha georgiana*) as the data revealed high genetic divergence between the two units.

*Plagiorhegma dubium* Maxim. is a rare myrmecochorous herb with a narrow distribution: Korea, northeast China, and eastern Siberia (Hutchinson, 1920). Myrmecochory is a mutualistic ant–plant interaction, where ants predate elaiosomes (fleshy structures attached to plant seeds) and disperse seeds (Bronstein, Alarcón, & Geber, 2006). Myrmecochory plays an important role for population growth and largely influences spatial distribution of genetic variation in obligatory myrmecochorous plants due to its substantial contribution to the plant migration (Pascov et al., 2015). Because ants disperse plant seeds over short geographic distances (less than 180 m at maximum; Gomez & Espadaler, 2013), gene flow among populations might be extremely limited with the limited migration. In Korea, *P. dubium* was rarely found in natural habitats and it is listed as an “endangered species” by the Ministry of Environment of South Korea in part due to limited migration ability as a myrmecochorous plant (Ministry of Environment, 2005). The species only inhabits dense deciduous forests mostly along the north side of shady valleys (Ghimire & Heo, 2012; Rhie, Lee, & Kim, 2015). However, the species was removed from the list in 2012 based on the recent population growth observed throughout the nation within the past 10 years (3rd National Environmental Survey, Ministry of Environment unpublished data; Suh & Kim, 2012). There is an urgent need of genetically based diagnosis for the possible biases from the survey of census population size and the expanded distribution in *P. dubium*.

In the present study, we aim to address (a) the level of genetic diversity in *Plagiorhegma dubium*; (b) how the species is genetically structured over heterogeneous landscapes; (c) the historical and contemporary gene flow; and (d) whether there have been recent and/or historical population bottlenecks. Using microsatellite markers, we investigated genetic diversity patterns of *P. dubium* throughout the global distribution (northeast Asia and Siberia). Due to rarity of the species, lower within‐population genetic diversity is expected. We hypothesize that populations of *P. dubium* may be largely structured over varying landscapes with limited migration rates through myrmecochory. Given that the species has not been commonly found until recently (within a few generations assuming generation time of 4 years; Smith, Ronsheim, & Swartz, 1986), we also hypothesized that the population expansion was a recent event after a series of historical bottlenecks.

## 2 MATERIALS AND METHODS

### 2.1 Study species

**Figure 1:**
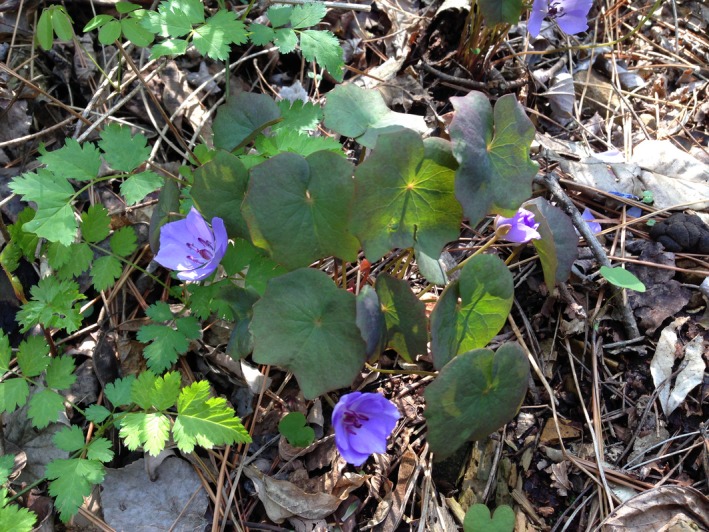
A photograph image of *Plagiorhegma dubium*. The photograph was taken by one of the author, Bo‐Yun Kim on the day, April 15, 2015 in Daegu, Gyeongbuk, S. Korea [Colour figure can be viewed at http://wileyonlinelibrary.com]

*Plagiorhegma dubium* Maxim. (Figure 1) is an herbaceous perennial that belongs to family Berberidaceae (Kim, Lee, Kim, & Kim, in press; Rhie et al., 2015). *P*.* dubium* is the only species in the genus *Plagiorhegma* Maxim. since the first discovery of the species (Maximowicz, 1859). Later, the species was moved to genus *Jeffersonia* Bart. due to its similarity in floral structure with the species in *Jeffersonia* (*J. diphylla* (L.) Perss; Bentham & Hooker, 1862; Ying, Boufford, & Brach, 2008). The two species share several common features in morphology and are genetically closely related (Kim & Jansen, 1996; Kim, Kim, Kim, & Jansen, 2004). Despite the conflicts of taxonomic position of the species, in the present study, we will refer to it as *Plagiorhegma dubium* following the plant list (http://www.theplantlist.org/). Plants normally flower in the early spring around March to April and produce capsule fruits once a year (Kim, in press; Rhie et al., 2015). Flowers of the plant have both male and female parts in a single flower with six stamens and one carpel (Ghimire & Heo, 2012). Pollination of *P. dubium* is not yet surveyed; however, the species is likely pollinated by insects as the most closely species, *J. diphylla* is an insect‐pollinated species (Smith et al., 1986). There is a hypothesis that the species may be selectively autogamous to ensure seed set when there are only limited pollinators in cold springs (Smith et al., 1986). *P. dubium* may also vegetatively spread through rhizomes under harsh conditions (Smith et al., 1986). Due to a showy light violet flower and heart shape leaves in mature plants, about 10–30 cm long, the plants have been recognized as a potential ornamental species (Rhie et al., 2015; Song, 2007). The species also has been well known for its medicinal uses. The plant roots have been used as a folk medicine for stomach aches and as phytochemical extract called “berberine” proven to be effective in lowering cholesterol (Bae, 2000; Kong et al., 2004).

### 2.2 Sample collection and DNA isolation

**Figure 2:**
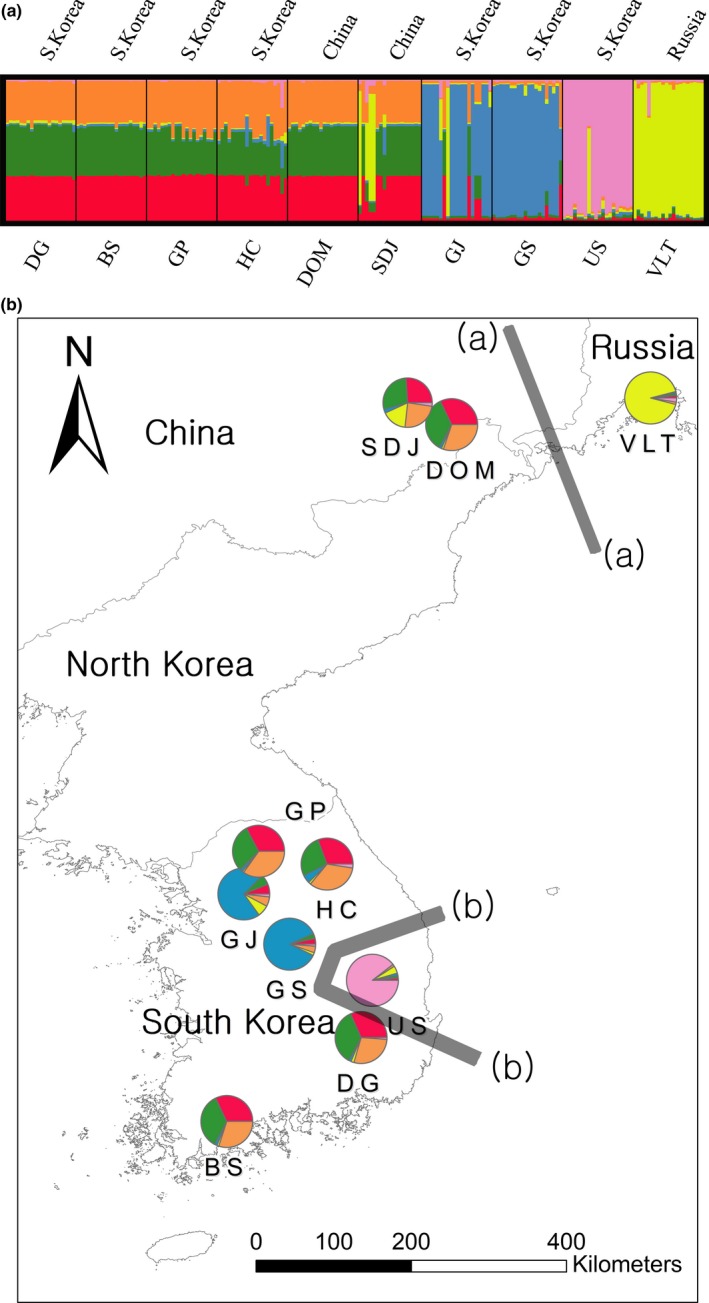
Bayesian model‐based clustering analysis for 10 *Plagiorhegma dubium* populations. (a) Bar plot presents group assignments for 198 individual genotypes for the optimal number of clusters *K *=* *6 (Δ*K* = 343.17; Supporting Information Figure S1). Populations are separated by the vertical black lines. (b) Pie charts on the location map with first two ranked barriers identified by Barrier analysis. The pie charts show the frequency of each cluster in a population based on STRUCTURE results. The size of pies is proportional to sample size. See Table 1 for abbreviation of population locations and sample sizes. Genetic barriers are depicted by gray lines with alphabetically ranked orders. Robustness of each barrier was estimated by 100 bootstraps and represented by the thickness of each line (barrier a > 75% support; barrier b > 95% support). The remaining barriers were not present as statistical significances are very weak at a support level of <50% [Colour figure can be viewed at http://wileyonlinelibrary.com]

Sample collections were made in two consecutive years (2014 and 2015). We collected samples throughout the global distribution (Korea, northeast China, eastern Siberia) except for North Korea due to the inaccessibility. For the seven Korean domestic populations, we randomly chose collection sites: the distances between domestic populations in South Korea vary from 30 km for the closest population pairs to 150 km for the most distant population pairs (Figure 2). Most Korean populations were patchy and the population sizes ranged from 40 m × 40 m to 100 m × 150 m with ~ 500 individuals or less. In contrast, Chinese populations were relatively high in density (e.g., ~1,000 individuals within ~200 m × 50 m area for Sandaowanzhen). We collected young leaves of 198 individuals from 10 populations (Table 1). Although empirically tested rates of clonal propagation are unknown, it is likely that the species can propagate clonally under harsh environmental conditions (Smith et al., 1986). To avoid collecting multiple samples from clones, we put at least 1.5 m distances among individuals within each population. We obtained all required permits to collect samples in protected areas from the Korean National Park Service under related rules and policies. Vouchers of collections (KNR2015110–KNR2015250; KNR2016061–KNR2016140) were deposited in Hallym University herbarium. Leaf tissues collected were preserved at room temperature in plastic bags with silica‐gel desiccants until DNA extraction. We extracted genomic DNA from the dried tissues using DNeasy Plant Mini Kit (Qiagen, Hilden, Germany) following the manufacturer's protocol. Quantity and quality of DNA isolates were assessed using a NanoDrop ND1000 (Thermo Fisher Scientific; quality cutoff, OD 260/280 ratio between 1.7 and 1.9) and visualized using a 1.5% agarose‐gel electrophoresis.

**Table 1:** Summary of genetic diversity indices and location information of collection sites for *Plagiorhegma dubium*

Location	Abbreviation	*N*	Lon	Lat	He (±*SE*)	Ho (±*SE*)	Na (±*SE*)	Ne (±*SE*)
Uiseong, Gyeongbuk, S. Korea	US	20	128.774	36.470	0.31 (0.07)	0.34 (0.12)	1.9 (0.13)	1.5 (0.13)
Daegu, Gyeongbuk, S. Korea	DG	20	128.643	35.808	0.21 (0.09)	0.38 (0.17)	1.5 (0.19)	1.3 (0.17)
Boseong., Jeonnam, S. Korea	BS	20	127.086	34.835	0.14 (0.08)	0.26 (0.16)	1.5 (0.19)	1.3 (0.16)
Gwangju, Gyeonggi, S. Korea	GJ	20	127.283	37.469	0.20 (0.08)	0.27 (0.15)	1.9 (0.23)	1.4 (0.15)
Goesan, Chungbuk, S. Korea	GS	20	127.815	36.888	0.32 (0.09)	0.42 (0.14)	2.0 (0.27)	1.6 (0.18)
Gapyeong, Gyeonggi, S. Korea	GP	20	127.455	37.964	0.23 (0.08)	0.36 (0.14)	1.6 (0.18)	1.4 (0.16)
Hongcheon, Gangwon, S. Korea	HC	20	128.249	37.816	0.24 (0.08)	0.34 (0.14)	1.8 (0.25)	1.4 (0.16)
Tumen, Jilin, China	DOM	20	129.693	42.908	0.14 (0.07)	0.20 (0.11)	1.5 (0.19)	1.3 (0.13)
Sandaowanzhen, Jilin, China	SDJ	20	129.182	43.162	0.18 (0.08)	0.26 (0.16)	1.5 (0.19)	1.3 (0.16)
Vladivostok, Russia	VLT	18	132.000	43.217	0.26 (0.11)	0.46 (0.11)	2.1 (0.23)	1.6 (0.16)

*Note*

N: sample size; Lon & Lat: geographic coordinates; He & Ho: mean expected and observed heterozygosity for each of 10 populations; Na & Ne: mean number of alleles and allelic richness for each of 10 populations; *SE*: standard error.

### 2.3 Microsatellite genotyping

Fourteen microsatellite loci were used to genotype the total of 198 individuals from 10 populations using primers developed and tested for *Plagiorhegma dubium* (Kim et al., in press; see Table 1 for the sample information). We performed PCRs in a volume of 25 μl containing 1 ul of 10 ng template DNA, 2 μl dNTPs (20 mM), 2.5 μl 10 × PCR buffer containing MgCl_2_ (Takara, Japan), fluorescently labeled (FAM, HEX, and NED) forward and reverse primers, 0.5 μl (10 pmol each). PCR cycling conditions were as follows: 5 min predenaturation at 95°C followed by 30 cycles of 1 min at 95°C, 1 min at 50°C, and 1 min at 72°C, followed by a final 10 min extension step at 72°C. The fluorescently labeled PCR products were pooled with a size standard GS500LIZ (Applied Biosystems, USA). ABI 3730XL automated sequencer (Applied Biosystems) was used to separate out the amplified fragments. Using Gene Marker program (version 2.40, Softgenetics LLC), we examined microsatellite profiles with automated scoring. We then manually checked the result to avoid scoring errors. We tested for presence of null alleles using FreeNA for each of 14 loci within each population (Chapuis & Estoup, 2007).

### 2.4 Data analysis

We tested whether there is a significant deviation from Hardy–Weinberg equilibrium (HWE) and linkage disequilibrium (LD) within each population for each of the 14 microsatellite markers using Fisher's exact test (Guo & Thompson, 1992) in Arlequin v. 3.5 (Excoffier & Lischer, 2010). Bonferroni corrections were used to adjust for significant alpha (*p*) values for HWE and LD. Six of 14 markers were with moderate to high frequency of null alleles in more than two populations. The six markers were either significantly departure from HWE or not independent from one another (not in LD *r* > 0.5; *p* < 0.0001) in over two or more populations. Some of statistical analyses such as STRUCTURE depend largely on two strong assumptions, that is, Hardy–Weinberg equilibrium (HWE) and marker independence within populations (Pritchard, Stevens, & Donnelly, 2000). Because significant deviation from HWE is often linked to heterozygote excess or deficiency, the six loci would also have great impact not only on genetic diversity pattern but also genetic distance‐based genetic analyses, for example, PCoA and Mantle test. Therefore, we only used eight microsatellite markers for all downstream analyses to avoid marker‐based biases. We performed clonality test to avoid presence of multiple clones within each population for genetic analyses. Identical multilocus genotypes (MLG) can be resulted from clonal propagation or distinct sexual reproduction events by chance. We used GenClone v. 2.0 (Arnaud‐Haond & Belkhir 2007) to determine true clonal ramets in our samples. GenClone was performed for each population separately with only polymorphic loci included. The probability of identical MLG due to sexual reproduction by chance (PgenF_IS_) was calculated in each population (cut off for PgenF_IS_ < 0.05).

We calculated the following genetic diversity measures: expected & observed heterozygosity (He & Ho), number of alleles (Na) allelic richness (Ne), and inbreeding coefficient (*F*_IS_) using Arlequin v. 3.5 and GENALEX v. 6.5 (Excoffier & Lischer, 2010; Peakall & Smouse, 2012). We estimated pairwise *F*_ST_ between population pairs across all 10 populations in Arlequin v. 3.5. For significance testing, we applied 1,000 permutations with replacements. Slatkin (1995) suggested to use R_ST_ for highly variable microsatellite markers assuming the mutation rate is strictly under a generalized stepwise mutation model. Given that microsatellites hardly are under a strict stepwise mutation model in practice, R_ST_ is not reliable and even prone to biases (Balloux & Goudet, 2002; Lugon‐Moulin, Brunner, Wyttenbach, Hausser, & Goudet, 1999; Meirmans & Hedrick, 2011). Therefore, we estimated and used *F*_ST_ in the present study.

Population genetic structure was investigated using a Bayesian model‐based clustering approach implemented in STRUCTURE v. 2.3.4 (Pritchard et al., 2000). We used the admixture for ancestry model and the correlated allele frequency model (Falush, Stephens, & Pritchard, 2003). Ten independent runs were repeated with 1,000,000 MCMC iterations following 100,000 steps of burn‐in for each *K* from 1 to 12. To infer the number of clusters (*K*) best explaining the data, Δ*K* was calculated (Evanno, Regnaut, & Goudet, 2005) using STRUCTURE HARVESTER Web v. 0.6.94 (Earl & vonHoldt, 2012). Ancestry coefficients of the individual genotypes from 10 repeated STRUCTURE runs were summarized using CLUMPP v. 1.1.2 (Jakobsson & Rosenberg, 2007) with greedy option. We visualized the result in DISTRUCT v. 1.1 (Rosenberg, 2004). Using Monmonier's (1973) maximum difference algorithm implemented in Barrier v 2.2 (Manni, Guerard, & Heyer, 2004), we identified significant genetic discontinuities between groups of populations. We calculated Nei's genetic distances (Nei, Tajima, & Tateno, 1983) for Barrier analysis. We estimated 100 distance matrices by bootstrapping over loci in MICROSATELLITE ANALYSER (MSA) v. 4.05 (Dieringer & Schlotterer, 2003). The robustness of barriers was examined using the bootstrapped distance matrices. To investigate the pattern of clustering among individual genotypes, we also performed principal coordinate analysis (PCoA) on Nei's genetic distance estimated for all 198 individuals in GENALEX v. 6.5. We conducted Analysis of molecular variance (AMOVA) in Arlequin v. 3.5 to hierarchically partition the total molecular variance within and between groups identified by STRUCTURE analysis. We compared the values against 1,000 resampled data sets to determine statistical significance.

We performed a Mantel test to assess isolation by distance (IBD) based on pairwise genetic distance (Slatkin's linearized *F*_ST_ = *F*_ST_/(1 − *F*_ST_)) and log‐transformed Euclidean distance for all population pairs (Rousset, 1997) in GENALEX v. 6.5. One thousand random permutations with replacement were used to examine if the correlation coefficient (*r*) between the pairwise *F*_ST_ and the Euclidean distance was significantly different from zero.

We estimated historical migration rates among populations and long‐term effective population sizes using MIGRATE‐N 3.6.11 (Beerli, 2008). MIGRATE‐N employs coalescent approach to estimate both effective population size θ (4Neμ, where Ne = effective population size; *μ* = mutation rate) and asymmetric migration rate, *M* (M = *m*/*μ*, where *m* = immigration rate/generation) between populations over a long time scale (Beerli & Felsenstein, 2001). We used Bayesian approach and ran two replicates of MIGRATE‐N with the Brownian motion mutation model. We chose uniform prior distributions for *θ* estimation (*θ*: 0–100, *M*: 0–100). To reduce the computational challenges, we reformed groups based on the genetic clusters identified by STRUCTURE (Figure 2; 4 groups, cluster1 = DG, BS, GP, HC, DOM, and SDJ; cluster2 = GJ, GS; US; VLT; see Table 1 for abbreviations). We also estimated contemporary migration rate among the same groups used in MIGRATE‐N over the last few generations in BAYESASS 3.0.1 (Wilson & Rannala, 2003). BAYESASS was started with a 1,000,000 burn‐in followed by 10,000,000 MCMC iterations using a sampling frequency of 2,000. We used the default setting for mixing parameters. To compare historical and contemporary migration rates, migration rates estimated from MIGRATE‐N were adjusted by mutation rate, 10^−4^/allele/generation found in Marriage et al. (2009) for microsatellite markers in *Arabidopsis thaliana* (Chiucchi & Gibbs, 2010). We performed a nonparametric hypothesis testing to assess whether difference between the two migration rates is statistically robust using permutation test with 1,000 replicates in R 3.4.4 (R Core Team 2018).

Historical population bottlenecks were examined using Garza–Williamson index (G‐W index; M‐ratio) implemented in Arlequin v. 3.5 (Excoffier & Lischer, 2010). The M‐ratio test (Garza & Williamson, 2001) uses the ratio of the number of alleles to the allele size range because a bottleneck is expected to reduce allele number faster than the range of allele sizes. The M‐ratio values were statistically tested by 10,000 simulated replicates assuming an effective size, 40 (2 × average sample size per population; 2 was used for diploid) in Critical_M (Garza & Williamson, 2001; available at https://swfsc.noaa.gov/textblock.aspx?Division=FED&id=3298). We also estimated relatively recent reduction in population size within the past few generations using BOTTLENECK v. 1.2.02 (Cornuet & Luikart, 1996; Piry, Luikart, & Cornuet, 1999). BOTTLENECK infers population bottlenecks based on a significant excess or deficit of heterozygosity relative to equilibrium state (Piry et al., 1999). According to a simulation study, the M‐ratio is well equipped for identifying a historical bottleneck whereas BOTTLENECK is better suited for detecting recent bottleneck events (Williamson‐Natesan, 2005). BOTTLENECK was run under infinite allele model (IAM) and stepwise mutation model (SMM) with sign and Wilcoxon's sign rank test for statistical significance. We also investigated whether there is mode shift from equilibrium state in allele frequencies.

## 3 RESULTS

No scoring errors were identified during the genotyping procedures. We found null alleles in six of the 14 microsatellite loci with the frequency of minor to moderate (0.1–0.3 in loci JD_27, JD_66, JD_77, JD_78, JD_81, and JD_93) in more than two populations. After filtering the six microsatellite markers with null alleles, all 8 markers did not deviate from HWE nor were in LD after Bonferroni correction (*p* > 0.05). We found significant excess of heterozygotes in those 6 loci (observed heterozygosity > 0.7). Two of the six loci for three populations were with moderate (0.13) to high frequency (0.30) of null alleles. There were a few identical MLGs found in two populations (DG and GJ). However high values of PgenF_IS_ (average = 0.71) indicate that those identical MLGs were not true clones. Therefore, we treated all individual samples were not clones for the subsequent analysis. The mean expected heterozygosity per population ranged from 0.14 in BS (see Table 1 for population abbreviations) to 0.32 in GS, whereas the mean observed heterozygosity varied from 0.1 (DOM) to 0.46 (VLT) (Table 1). The mean expected heterozygosity and the observed heterozygosity largely differ from one another (*t* = 5.45, *df* = 9, *p* < 0.01; one tail *t* test with unequal variance). The mean number of alleles for each population and allelic richness were the highest in VLT (Na = 2.1; Ne = 1.6), whereas the lowest allelic richness was shown by populations BS, DG, DOM, and SDJ (Na = 1.5; Ne = 1.3; Table 1). To investigate effects of those six loci with heterozygote excess that also showed presence of null alleles, we additionally estimated the genetic diversity parameters with all 14 markers. The results with 14 markers clearly had higher genetic variation in heterozygosity and allele numbers (average He_14_ = 0.37 He_8_=0.22; average Ho_14_ = 0.59 Ho_8_ = 0.33; average Na_14_ = 2.0 Na_8_ = 1.7; average Ne_14_ = 1.7 Ne_8_ = 1.4; Table 1). We provided genetic diversity estimated for each of the eight loci as Supporting Information (Table S1). We did not find any positive values of *F*_IS_ significantly different from zero in all populations; therefore, we do not present *F*_IS_ values here. Pairwise *F*_ST_ values were constantly higher between US and the other 9 populations (mean *F*_ST_ = 0.44) than the rest of the population pairs (mean *F*_ST_ = 0.24; Table 2). Pairwise *F*_ST_ values were also high when population VLT was compared to all other (average *F*_ST_ = 0.40; Table 2).

**Table 2:** Estimated pairwise *F*_ST_ (below diagonal) values among 10 *Plagiorhegma dubium* populations throughout East Asia and Russia

	US	DG	BS	GP	HC	DOM	SDJ	GJ	GS	VLT
US	0.000	—	—	—	—	—	—	—	—	—
DG	0.463	0.000	—	—	—	—	—	—	—	—
BS	0.513	0.086	0.000	—	—	—	—	—	—	—
GP	0.456	0.110	0.066	0.000	—	—	—	—	—	—
HC	0.414	0.148	0.086	0.014 ns	0.000	—	—	—	—	—
DOM	0.519	0.106	0.036	0.061	0.099	0.000	—	—	—	—
SDJ	0.450	0.119	0.012ns	0.091	0.109	0.058	0.000	—	—	—
GJ	0.441	0.322	0.323	0.314	0.293	0.335	0.291	0.000	—	—
GS	0.371	0.324	0.318	0.250	0.196	0.324	0.312	0.124	0.000	—
VLT	0.284	0.397	0.464	0.430	0.421	0.453	0.373	0.423	0.397	0.000

*Note*

See Table 1 for abbreviation of population locations and sample sizes. All values estimated were significantly different from 0 at the *p *<* *0.05 level with Bonferroni correction except for two values marked with ns.

**Figure 3:**
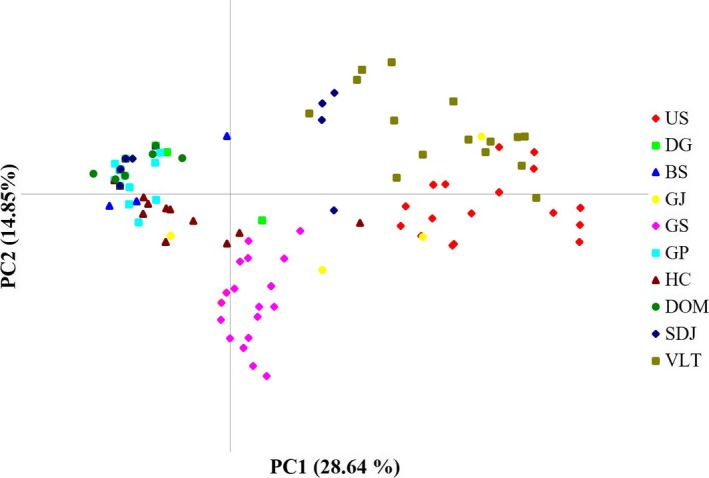
Principle components analysis plot of 198 *Plagiorhegma dubium* individuals from 10 populations. The first two variance components from all eight microsatellite loci were plotted. See Table 1 for abbreviation of population locations and sample sizes [Colour figure can be viewed at http://wileyonlinelibrary.com]

**Figure 4:**
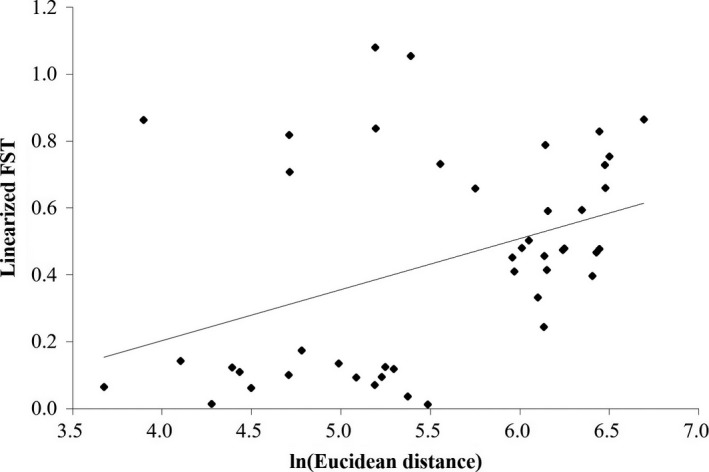
The association between the logarithm of Euclidean distance (km) and Slatkin's linearized *F*_ST_ (*F*_ST_/(1 − *F*_ST_)) for all possible population pairs of 10 *Plagiorhegma dubium* populations from the northeast Asia and Siberia. A Mantel test showed a significant Isolation by Distance pattern (*r* = 0.4, *p* < 0.05)

Based on delta *K* from STRUCTURE results, the number of clusters (*K*, number of randomly mating subgroups) that best explain the data was 6 (Supporting Information Table S2 and Figure S1). To show the clustering pattern with various number of *K* groups, we provided bar plots of *K* = 2–8 (Supporting Information Figure S2). The populations US and VLT were the most genetically distinct, followed by GS and GJ (Figure 2a,b). Four populations (BS, DG, GP, and HC) in South Korea shared alleles with two populations located in northeast China (DOM, SDJ; Figure 2a,b). Results of the Barrier analysis identified strong genetic discontinuity between the VLT and US and all the other 8 populations by the first and the second rank barriers with over 75% and 95% bootstrap support, respectively (Figure 2b). The remaining barriers (third to sixth barriers) were not presented due to low bootstrap support (≪ 50%). Post hoc AMOVA of the groups identified by both STRUCTURE and Barrier analyses (four groups: US, VLT, GS, and GJ, the other six populations) showed that populations were more genetically differentiated across groups (*F*_CT_ = 0.50) than within groups (*F*_SC_ = 0.16; Table 3). AMOVA also revealed that the most genetic variance was partitioned to among group variation (50%) followed by within individuals (39%; Table 3). Consistent with the STRUCTURE result, the PCoA largely separated out US and VLT from the other 8 populations on the first PC axis (PC1, explained 29% of molecular variance; Figure 3). GS population was forming a cluster that was differentiated from the rest of populations particularly on the second axis (PC1, explain 15% of molecular variance; Figure 3). Genotypes in GJ were scattered throughout both axes with some level of overlap with GS (Figure 3). Results of Mantel test showed a significant correlation between genetic divergence (*F*_ST_) and geographic distance (Euclidean distance; *r* = 0.4, *p* < 0.05; Figure 4).

**Table 3:** Analysis of molecular variance (AMOVA) results of 10 *Plagiorhegma dubium* populations within four regions identified by STRUCTURE (US; GP&GS; VLT; the other six populations; Figure 1; See Table 1 for population abbreviations)

Source	Sum of squares	Variance components	Percentage of variation	Fixation index
Among groups (*F*_CT_)	132.10	0.54	50.00	0.50
Among population within groups (*F*_SC_)	22.41	0.08	7.80	0.16
Among individuals within populations (*F*_IS_)	89.38	0.03	3.00	0.07
Within individuals (*F*_IT_)	82.50	0.42	39.24	0.61

*Note*

All variance components were statistically significant (*p* < 0.01).

There was no evidence of asymmetric migration based on the overlapping values between the two directions in 95% confidence intervals for both long‐term and contemporary migration rates (CI; Supporting Information Table S3). All contemporary migration rates among four groups estimated from BAYESASS overlapped 0 at the 95% CI, suggesting very little or no recent migration (Supporting Information Table S3). The long‐term migration rates (*M*_H_) of MIGRATE‐N ranged from 0.184 (US to CL1) to 4.65 (CL1 to VLT; Supporting Information Table S3). After adjusting for the mutation rate, M_H_ values for all cluster pairs were much lower than the M_C_ values and the difference was statistically significant (*p* < 0.01; Supporting Information Table S3). The estimated θ in MIGRATE‐N analysis ranged from 0.33 to 1.26, which are comparable to the ones observed in a long‐lived shrub, *Rhododendron* spp. (Hsieh et al., 2013) and an annual *Helianthus* spp. (Kane et al., 2009). The long‐term mutation‐scaled effective population size was the smallest in US (814, 95% CI 250–4,500) and the largest in the cluster2, a group including population GJ and GS (3160, 95% CI 3150–7,500), with the mutation rate.

From the results of the BOTTLENECK analysis, we did not find evidence of recent population bottlenecks for most populations under both IAM and SMM mutation models from the two significant tests except for US (Table 4). G‐W indices (M) of all 10 populations, on the other hand, were lower than the critical value of *M* = 0.73, estimated from data (Table 4).

**Table 4:** Results of tests for the recent and past bottlenecks in *Plagiorhegma dubium* populations: G‐W index is the Garza–Williamson index known as M‐ratio, the ratio of number of alleles to allele size range

Population	G‐W index (±*SD*)	*p* (Sign test)		*p* (Wilcoxon test)		Mode shift
		IAM	SMM	IAM	SMM	
US	0.60 (0.09)	0.03^a^	0.05^a^	0.04^a^	0.11	Yes
DG	0.58 (0.10)	0.20	0.30	0.13	0.13	Yes
BS	0.58 (0.10)	0.56	0.65	0.31	0.31	Yes
GJ	0.51 (0.19)	0.25	0.36	0.69	0.84	No
GS	0.50 (0.16)	0.08	0.14	0.08	0.11	Yes
GP	0.52 (0.09)	0.10	0.16	0.09	0.09	Yes
HC	0.58 (0.10)	0.14	0.20	0.63	0.63	No
DOM	0.58 (0.10)	0.56	0.66	0.31	0.88	Yes
SDJ	0.53 (0.14)	0.22	0.34	0.13	0.19	Yes
VLT	0.56 (0.12)	0.41	0.48	0.47	1.00	No

*Notes*

*p*‐Values from sign and Wilcoxon signed‐rank tests for excess or deficit of heterozygosity under IAM and SMM mutation models. Mode shift—to check whether the allele frequency distribution to distorting L‐shape distribution indicating recent bottlenecks.

a Statistical significance at the *p *<* *0.05 level.

## 4 DISCUSSION

Most rare and threatened plants may severely suffer from erosion of genetic diversity, inbreeding depression and limited gene flow derived by reduced population size and/or fragmented habitats (Ellstrand & Elam, 1993; Ottewell et al., 2015; Young et al., 1996). Conservation and management plans for threatened plant species could benefit from genetic considerations (Hedrick, 2001). There are several ways to take advantage of genetic data for conservation including the followings: (a) diagnose the status of genetic erosion; (b) infer changes in demography; and (c) identify evolutionarily significant units. Our genetic analyses suggested that *Plagiorhegma dubium* has historically experienced population bottlenecks despite the recent population growth. We found two genetically distinctive groups (a population US and a group with GS, GJ) with a unique pattern of genetic diversity, which might be an important unit for conservation in Korea.

Significant departures from HWE associated with significant excess of heterozygotes were found over half of 10 populations for six of fourteen microsatellite loci. The heterozygote deficiency was minor and can be explained by the presence of null alleles. In fact, we found moderate to high frequency of null alleles in two of those 6 loci for three populations. The excess of heterozygotes was apparent, however, unlike the heterozygote deficiency, only a few explanations have been suggested for the excess: small sexual & self‐incompatible populations, over dominance, negative assortative mating and asexual reproduction (Stoeckel et al., 2006). Given that biological and ecological features of *P*. *dubium* are largely unknown, we are open to all four explanations. Future studies may build up basic understandings of the species from our study and examine the hypotheses with properly designed experiments. For example, as in Stoeckel et al. (2006), the most likely cause of heterozygote excess in *P*. *dubium* may be examined by incorporating both molecular analysis and breeding experiments with variety of sample sizes.

We found inflation of genetic variation when using all 14 loci (average He_14_ = 0.37; Ho_14_ = 0.59; Na_14_ = 2.0; Ne_14_ = 1.7) relative to the one with 8 loci that didn't show excess of heterozygote and/or presence of null alleles (average He_8_ = 0.22; Ho_8_ = 0.33; Na_8_ = 1.7; Ne_8_ = 1.4). Those six loci screened out were nearly fixed for heterozygotes in most of the 10 populations, which makes the two alternate alleles equally frequent resulting in high expected heterozygosity (the maximum He = 0.5, when *p* = 0.5). Given that most of the loci only showed 1–3 alleles per population, the six loci with heterozygote excess also were contributing greatly to variation in allele numbers.

Consistent with our hypothesis, within‐population genetic variation of *P*.* dubium* was lower (mean He = 0.22; mean Ho = 0.33; Table 1) than the ones found in short‐lived perennials measured from microsatellite data (He = 0.55; Ho = 0.53; Nybom, 2004). Although genetic variation is highly related to breeding systems in plants (Baker, 1959; Barrett, 2003; Charlesworth, 2003), the reduced genetic variation in our study may not be attributable to inbreeding. *F*_IS_ values were low and even negative in several populations for a few loci, which suggest no indication of inbreeding. In fact, high heterozygosity and low inbreeding rates are often linked with self‐incompatible outcrossing systems (Baker, 1959; Barrett, 2003; Charlesworth, 2003). For example, *Ulmus pumila*, one of self‐incompatible elm species showed low inbreeding rate with high heterozygosity (Zalapa, Brunet, & Guries, 2010). However, unlike many self‐incompatible outcrossing plant species, we found reduced heterozygosity in *P*. *dubium*. One explanation for the low genetic variation would be recent and/or historical population bottlenecks. Despite the moderate to large long‐term effective population sizes estimated from MIGRATE‐N (814‐3160), M ratios (G‐W index) appeared to be lower than the threshold (M = 0.73) estimate from Critical_M in all 10 populations. It suggests that there were drastic changes in population sizes over a long period of time. Although *P*. *dubium* is autogamous allowing selfing (Smith et al., 1986), low inbreeding coefficient values preclude the hypothesis of inbreeding as the causal mechanism for the reduced genetic variation. Thus, in *P*. *dubium*, historical population bottlenecks are the likely cause of reduction in genetic diversity.

Distribution of alleles and genotypes across space and time is not random but the result from joint effects of several evolutionary forces; that is, mutation, selection, migration, and stochasticity (Loveless & Hamrick, 1984; Spieth, 1974). Therefore, mating system, migration, and ecological factors associated with both elements greatly influence the genetic structure of plants (Loveless & Hamrick, 1984). The average among‐population divergence of *P*.* dubium* (*F*_ST_ = 0.24) was comparable but not significantly higher than the ones found in similar life form plant species (*F*_ST_ = 0.31 estimated from microsatellite data; Nybom, 2004; Table 2). It is somewhat conflicting to our hypothesis given the biology of the species. *P*. *dubium* is insect pollinated with the capability of self‐fertilization and vegetative propagation (Smith et al., 1986). Seeds are migrated mostly by multiple ant species (Jo, Kim, Seo, & Lee, 2015; Smith et al., 1986). As seeds are migrated over very limited geographic distances (Giladi, 2006; Horvitz & Schemske, 1986), populations are expected to be highly structured in most myrmecochorous species. Furthermore, the species has been considered rare and endangered until recently, which suggests the populations are likely to be small and isolated. Given that the indirect measure of migration (*F*_ST_ estimates) assume rather unrealistic island model with no mutation, no selection and particularly equal population sizes, *F*_ST_ does not directly reflect migration and gene flow (Whitlock & McCauley, 1999). Indeed, both recent and long‐term migration rates we estimated were very low by overlapping or close to zero at 95% CI, indicating limited gene flow among groups (Supporting Information Table S3). The limited gene flow would have resulted in population isolation and creating the observed spatial structure. Alternatively, historical population bottlenecks found in all 10 populations might have influenced the spatial pattern of genetic structure (Loveless & Hamrick, 1984).

The population US and the group with GS and GJ were genetically distinct from the neighboring populations despite the geographic proximity (Figure 2a,b). PCoA and Barrier results revealed a similar pattern that separates the three populations from the neighboring populations (Figures 2 and 3). The abrupt shift of allele frequency distribution in the three populations may be due to environmental barriers that were not formerly recognized. It is more likely that demographic changes experienced in the past may be responsible for the spatial structure that do not reflect geographic distances (Table 4). Despite the complex spatial structures observed, there was a significant IBD pattern among the 10 populations (Figure 4). The significant IBD is somewhat consistent with the spatial pattern, which might reflect the limited gene flow over a large scale due to reduced migration capacity of the species by ants. Notably, Bottleneck results indicated a severe recent bottleneck in the most genetically distinct population, US (Table 4). Population bottlenecks are likely to fix different alleles for each local population resulting in rather random population structure that may not necessarily reflect geography. Since we couldn't confirm any signs of visual damages near the site, the cause of the bottlenecks may not be habitat destructions. Climate‐related factors associated with pollinator crashes are more likely the causes of the observed bottlenecks. Unfortunately, we couldn't determine the real causes of the recent bottleneck in US due to lack of monitoring information.

Without proper genetic considerations, *P*. *dubium* was cleared from the endangered species list in Korea based on recent growth in census population sizes and range expansion (Suh & Kim, 2012). Our research revealed that the species only harbors a limited amount of individual‐level genetic variation (Table 1). On top of that, the genetic diversity pattern clearly indicated that all populations have experienced recent and/or historical bottlenecks. Population genetics can offer insights for management plans of threatened plant species (Hedrick, 2001; Hughes et al. 2008; Aavik et al., 2017). Our findings may be applicable for practical conservation and management plans for *P*. *dubium*. First, US, GS, and GJ populations with unique alleles should be treated as separate units for conservation in Korea. Additionally, Chinese populations can be strong candidates for a restoration project in Korea since most Korean populations shared alleles and genotypes with Chinese populations. Although the *P*. *dubium* population has been growing and expanding in range with conservation efforts in Korean peninsula, the populations should be genetically monitored to avoid inaccurate evaluation.

However, the pattern of genetic diversity and its distribution examined here may be biased to some extent as the study could not investigate populations in North Korea due to inaccessibility. There is an urgent need to conduct ecological and evolutionary monitoring for *P*. *dubium* populations in North Korea. Also, the fate of the populations might heavily depend on genetic variation that is adaptive particularly in radically changing environments, which neutral genetic markers cannot capture. Adaptive genetic variation in phenotypic traits should be examined for the future studies. Our study provides one good example of how genetic considerations can be appreciated as much as other ecological and political factors in management and conservation plans for rare and threatened species.

## CONFLICT OF INTEREST

The authors declare no conflict of interest.

## AUTHOR CONTRIBUTIONS

YD designed the project and achieved supporting grant. Sample collections, laboratory work, and microsatellite genotyping were conducted by BY. SR wrote the manuscript and conducted genetic and related statistical analyses. All authors edited the manuscript and agreed to submit current version of manuscript.

## DATA ACCESSIBILITY

Microsatellite data are available in the DRYAD Digital Repository (https://doi.org/10.5061/dryad.d6396sd).
