# Supplementary material for: Genetic diagnosis of a rare myrmecochorous species, Plagiorhegma dubium (Berberidaceae): Historical genetic bottlenecks and strong spatial structures among populations
Source: Ecol Evol. 2018 Aug 7;8(17):8791–802. doi: 10.1002/ece3.4362 (PMC6157670; doi:10.1002/ece3.4362)
Supplement: Supplementary file 1 [file ECE3-8-8791-s001.docx]

Supplementary Information

Genetic diagnosis of a rare myrmecochorous species, *Plagiorhegma dubium* (Berberidaceae): historical genetic bottlenecks and strong spatial structures among populations

Authors: Soo-Rang Lee^1^, Bo-Yun Kim^1^, Young-Dong Kim^*2^

^------------------------------------------------------------------------------------------------^

^1^Multidisciplinary Genome Institute, Life Science Hall, Hallym University, Hallymdaehak-gil, Chuncheon-si, Gangwon-do, S Korea 24252

^2^Department of Life Sciences, Life Science Hall # 8311, Hallym University, Hallymdaehak-gil, Chuncheon-si, Gangwon-do, S Korea 24252

Table S1. Within-population genetic diversity parameters for each of 8 markers in 10 *Plagiorhegma dubium* populations. See Table 1 for population sizes and abbreviations.

| **Pop** | **Locus** | **Na** | **Ne** | **Ho** | **He** |
| --- | --- | --- | --- | --- | --- |
| **US** | **JD_11** | 2.000 | 1.819 | 0.684 | 0.450 |
|  | **JD_27** | 2.000 | 1.471 | 0.400 | 0.320 |
|  | **JD_50** | 2.000 | 2.000 | 1.000 | 0.500 |
|  | **JD_66** | 1.000 | 1.000 | 0.000 | 0.000 |
|  | **JD_77** | 2.000 | 1.882 | 0.050 | 0.469 |
|  | **JD_38** | 2.000 | 1.406 | 0.250 | 0.289 |
|  | **JD_78** | 2.000 | 1.699 | 0.263 | 0.411 |
|  | **JD_81** | 2.000 | 1.051 | 0.050 | 0.049 |
| **DG** | **JD_11** | 2.000 | 1.956 | 0.850 | 0.489 |
|  | **JD_27** | 1.000 | 1.000 | 0.000 | 0.000 |
|  | **JD_50** | 2.000 | 2.000 | 1.000 | 0.500 |
|  | **JD_66** | 1.000 | 1.000 | 0.000 | 0.000 |
|  | **JD_77** | 1.000 | 1.000 | 0.000 | 0.000 |
|  | **JD_38** | 2.000 | 2.000 | 1.000 | 0.500 |
|  | **JD_78** | 2.000 | 1.220 | 0.200 | 0.180 |
|  | **JD_81** | 1.000 | 1.000 | 0.000 | 0.000 |
| **BS** | **JD_11** | 2.000 | 1.051 | 0.050 | 0.049 |
|  | **JD_27** | 1.000 | 1.000 | 0.000 | 0.000 |
|  | **JD_50** | 2.000 | 2.000 | 1.000 | 0.500 |
|  | **JD_66** | 1.000 | 1.000 | 0.000 | 0.000 |
|  | **JD_77** | 1.000 | 1.000 | 0.000 | 0.000 |
|  | **JD_38** | 2.000 | 1.995 | 0.950 | 0.499 |
|  | **JD_78** | 2.000 | 1.051 | 0.050 | 0.049 |
|  | **JD_81** | 1.000 | 1.000 | 0.000 | 0.000 |
| **GJ** | **JD_11** | 1.000 | 1.000 | 0.000 | 0.000 |
|  | **JD_27** | 2.000 | 1.280 | 0.050 | 0.219 |
|  | **JD_50** | 2.000 | 1.980 | 0.900 | 0.495 |
|  | **JD_66** | 2.000 | 1.362 | 0.105 | 0.266 |
|  | **JD_77** | 1.000 | 1.000 | 0.000 | 0.000 |
|  | **JD_38** | 2.000 | 2.000 | 1.000 | 0.500 |
|  | **JD_78** | 2.000 | 1.051 | 0.050 | 0.049 |
|  | **JD_81** | 3.000 | 1.107 | 0.050 | 0.096 |
| **GS** | **JD_11** | 1.000 | 1.000 | 0.000 | 0.000 |
|  | **JD_27** | 1.000 | 1.000 | 0.000 | 0.000 |
|  | **JD_50** | 2.000 | 1.915 | 0.789 | 0.478 |
|  | **JD_66** | 3.000 | 1.989 | 0.263 | 0.497 |
|  | **JD_77** | 3.000 | 2.241 | 0.600 | 0.554 |
|  | **JD_38** | 2.000 | 2.000 | 1.000 | 0.500 |
|  | **JD_78** | 2.000 | 1.782 | 0.650 | 0.439 |
|  | **JD_81** | 2.000 | 1.051 | 0.050 | 0.049 |
| **GP** | **JD_11** | 2.000 | 1.105 | 0.100 | 0.095 |
|  | **JD_27** | 1.000 | 1.000 | 0.000 | 0.000 |
|  | **JD_50** | 2.000 | 1.923 | 0.800 | 0.480 |
|  | **JD_66** | 1.000 | 1.000 | 0.000 | 0.000 |
|  | **JD_77** | 2.000 | 1.471 | 0.300 | 0.320 |
|  | **JD_38** | 2.000 | 1.995 | 0.950 | 0.499 |
|  | **JD_78** | 2.000 | 1.882 | 0.750 | 0.469 |
|  | **JD_81** | 1.000 | 1.000 | 0.000 | 0.000 |
| **HC** | **JD_11** | 1.000 | 1.000 | 0.000 | 0.000 |
|  | **JD_27** | 1.000 | 1.000 | 0.000 | 0.000 |
|  | **JD_50** | 2.000 | 1.978 | 0.895 | 0.494 |
|  | **JD_66** | 1.000 | 1.000 | 0.000 | 0.000 |
|  | **JD_77** | 2.000 | 1.699 | 0.263 | 0.411 |
|  | **JD_38** | 2.000 | 1.923 | 0.800 | 0.480 |
|  | **JD_78** | 2.000 | 1.835 | 0.700 | 0.455 |
|  | **JD_81** | 3.000 | 1.119 | 0.056 | 0.106 |
| **DOM** | **JD_11** | 2.000 | 1.105 | 0.100 | 0.095 |
|  | **JD_27** | 1.000 | 1.000 | 0.000 | 0.000 |
|  | **JD_50** | 2.000 | 1.600 | 0.400 | 0.375 |
|  | **JD_66** | 1.000 | 1.000 | 0.000 | 0.000 |
|  | **JD_77** | 1.000 | 1.000 | 0.000 | 0.000 |
|  | **JD_38** | 2.000 | 1.995 | 0.850 | 0.499 |
|  | **JD_78** | 2.000 | 1.220 | 0.200 | 0.180 |
|  | **JD_81** | 1.000 | 1.000 | 0.000 | 0.000 |
| **SDJ** | **JD_11** | 1.000 | 1.000 | 0.000 | 0.000 |
|  | **JD_27** | 2.000 | 1.562 | 0.000 | 0.360 |
|  | **JD_50** | 2.000 | 1.991 | 0.933 | 0.498 |
|  | **JD_66** | 1.000 | 1.000 | 0.000 | 0.000 |
|  | **JD_77** | 1.000 | 1.000 | 0.000 | 0.000 |
|  | **JD_38** | 2.000 | 2.000 | 1.000 | 0.500 |
|  | **JD_78** | 2.000 | 1.117 | 0.111 | 0.105 |
|  | **JD_81** | 1.000 | 1.000 | 0.000 | 0.000 |
| **VLT** | **JD_11** | 2.000 | 1.995 | 0.950 | 0.499 |
|  | **JD_27** | 2.000 | 1.161 | 0.050 | 0.139 |
|  | **JD_50** | 2.000 | 1.358 | 0.313 | 0.264 |
|  | **JD_66** | 3.000 | 2.381 | 0.200 | 0.580 |
|  | **JD_77** | 3.000 | 1.227 | 0.100 | 0.185 |
|  | **JD_38** | 2.000 | 1.835 | 0.400 | 0.455 |
|  | **JD_78** | 1.000 | 1.000 | 0.000 | 0.000 |
|  | **JD_81** | 2.000 | 1.161 | 0.050 | 0.139 |

Table S2. Probability of K clusters estimated from 10 STRUCTURE runs summarized by Evanno et al. (2005).

| **K** | **Reps** | **Mean LnP(K)** | **Stdev LnP(K)** | **Ln'(K)** | **\|Ln''(K)\|** | **Delta K** |
| --- | --- | --- | --- | --- | --- | --- |
| 1 | 10 | -1728.000000 | 0.000000 | — | — | — |
| 2 | 10 | -1469.240000 | 1.400952 | 258.760000 | 205.560000 | 146.728790 |
| 3 | 10 | -1416.040000 | 0.980023 | 53.200000 | 46.510000 | 47.458086 |
| 4 | 10 | -1316.330000 | 0.794495 | 99.710000 | 254.810000 | 320.719472 |
| 5 | 10 | -1471.430000 | 198.236985 | -155.100000 | 363.440000 | 1.833361 |
| **6** | **10** | **-1263.090000** | **0.785211** | **208.340000** | **269.460000** | **343.169117** |
| 7 | 10 | -1324.210000 | 4.169053 | -61.120000 | 39.640000 | 9.508155 |
| 8 | 10 | -1345.690000 | 23.494938 | -21.480000 | 3.760000 | 0.160034 |
| 9 | 10 | -1370.930000 | 32.269354 | -25.240000 | 51.520000 | 1.596561 |
| 10 | 10 | -1344.650000 | 7.996979 | 26.280000 | 9.860000 | 1.232966 |
| 11 | 10 | -1328.230000 | 3.502396 | 16.420000 | 16.760000 | 4.785296 |
| 12 | 10 | -1328.570000 | 3.264302 | -0.340000 | — | — |

Table S3. Contemporary (M_C_) and historical (M_H_) migration rates between population pairs estimated from BAYESASS and MIGTARE-N respectively. CL1 and CL2 denote cluster1 including 6 populations (DG, BS, GP, HC, DOM and SDJ) and cluster2 having 2 populations (GJ, GS) with similar genetic composition based on STRUCTURE respectively (Fig. 1). See Table 1 for abbreviation. CI stands for confidence interval. Mh_ μ is historical migration rates adjusted by mutation rate of 10e^-4^.

| Group1 | Group2 | Mc [±95%CI] | Mh [95%CI] | Mh_μ |
| --- | --- | --- | --- | --- |
| CL1 | US | 0.003 [0.006] | 3.087 [0.450,17.940] | 0.00031 |
| CL2 | US | 0.008 [0.016] | 2.733 [0.130,12.450] | 0.00027 |
| VLT | US | 0.014 [0.027] | 1.202 [0.165, 10.400] | 0.00012 |
| US | CL1 | 0.014 [0.027] | 0.184 [0.005,1.890] | 0.00002 |
| CL2 | CL1 | 0.045 [0.037] | 0.806 [0.000,3.600] | 0.00008 |
| VLT | CL1 | 0.015 [0.027] | 0.192 [0.000,1.800] | 0.00002 |
| US | CL2 | 0.014 [0.027] | 0.567 [0.030, 4.950] | 0.00006 |
| CL1 | CL2 | 0.003 [0.006] | 0.926 [0.000,5.590] | 0.00009 |
| VLT | CL2 | 0.014 [0.027] | 0.239 [0.000,2.400] | 0.00002 |
| US | VLT | 0.018 [0.031] | 0.893 [0.130,7.650] | 0.00009 |
| CL1 | VLT | 0.006 [0.009] | 4.649 [1.710,25.350] | 0.00046 |
| CL2 | VLT | 0.016 [0.021] | 3.272 [0.585,16.200] | 0.00033 |


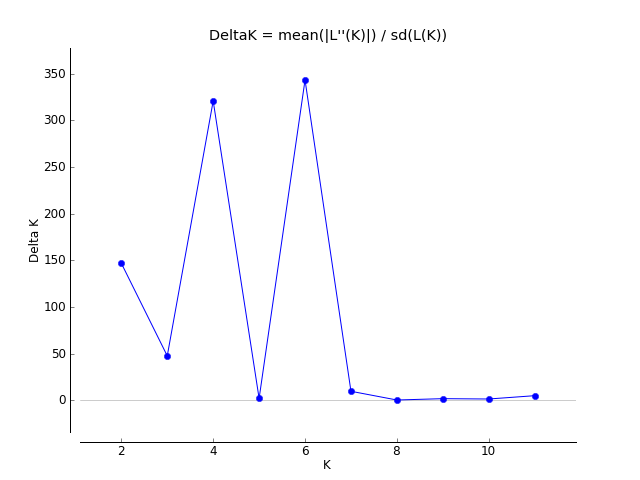


Figure S1: Delta K values for each of K clusters from 2 to 12 were estimated by method of Evanno et al. (2005).


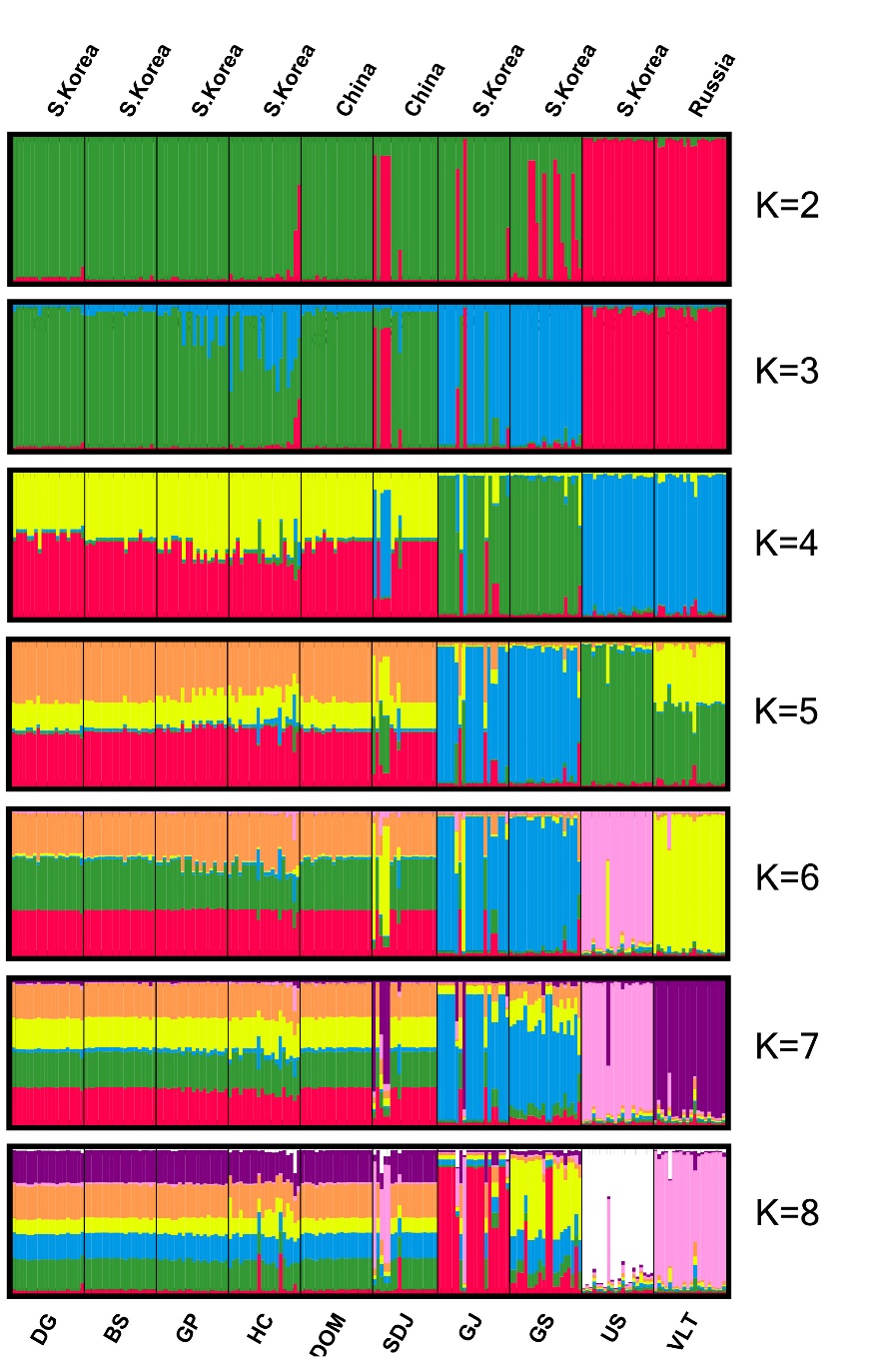


Figure S2: The Bayesian model-based group assignment for the *K* = 2 to *K* = 8 from STRUCTURE based on 8 unlinked microsatellite loci sampled across 198 individuals of *Plagiorhegma dubium*. Populations are separated by solid vertical black lines. Colors represent assignments of loci into each of the 2-8 estimated groups. See Table 1 for population abbreviations.
